# Web-Based Intervention and Email-Counseling for Problem Gamblers: Results of a Randomized Controlled Trial

**DOI:** 10.1007/s10899-019-09883-8

**Published:** 2019-09-27

**Authors:** Benjamin Jonas, Fabian Leuschner, Anna Eiling, Christine Schoelen, Renate Soellner, Peter Tossmann

**Affiliations:** 1 Delphi - Gesellschaft für Forschung, Beratung und Projektentwicklung mbH, Kaiserdamm 8, 14057 Berlin, Germany; 2 Federal Centre for Health Education (BZgA), Cologne, Germany; 3 University of Hildesheim, Hildesheim, Germany

**Keywords:** Pathological gambling, Problem gambling, Gambling disorder, Counseling, Email, Prevention

## Abstract

Web-based interventions have the potential to reduce the treatment gap for problem gambling. In the past years, several web-based help options were made available to the public. However, only few studies were conducted to test their effects. This study investigated the efficacy of two interventions for problem gamblers provided online by the German Federal Center for Health Education (BZgA). The first intervention is the guided program “Check Out” (CO), the second is email counselling (EC). A web-based randomized controlled trial with follow-up surveys after 3, 6 and 12 months was conducted. Participants were allocated to CO, to EC or to a waitlist (WL). Outcomes were the degree of problem gambling according to the Problem Gambling Severity Index, the number of days gambled in past 30 days, the highest stake during the past 30 days and the subjective well-being (WHO-5). 167 individuals were included in the trial. In comparison to the WL at the 3 months follow-up, participants of CO showed significant improvements with moderate to strong effect sizes in all outcomes. Strongest effects were found in the problem gambling severity (*d *= 0.91; *p *= 0.023), followed by the well-being (*d *= 0.70; *p *= 0.011), the gambling days (*d *= 0.59; *p *= 0.001) and the highest stake (*d *= 0.55; *p *= 0.012). Improvements were sustained until last follow-up. Compared to the WL, users of EC had beneficiary results in the problem gambling severity (*d *= 0.74; *p *= 0.022). No significant effect differences were found between CO and EC. However, according to process evaluation, users of CO reported a significantly stronger working alliance than users of EC (*d *= 0.70; *p *= 0.019) and used the intervention considerably longer (*d *= 0.84; *p *= 0.004). CO helps treatment-seeking individuals to sustainably reduce their gambling behavior and to increase their general well-being. Compared to EC, CO seems a better support option, since its effects include a wider range of outcomes. Possible reasons are the more engaging program structure and elements of CO, as well as the closer interaction between client and counselor.

## Introduction

Pathological gambling has substantial impact on the lives of gamblers and their relatives, and is associated with an elevated risk for several substance-related and other mental disorders (Langham et al. 2016; Meyer and Bachmann 2017). The past-year prevalences for problematic and pathological gambling in Germany amount to 0.8% (Haß and Lang 2016). In Europe, the past-year prevalence of problematic gambling varies between 0.1 and 3.4% (Calado and Griffiths 2016). Gaming machines, sports betting, casino- and online-gambling are strongly associated with pathological gambling (Sassen et al. 2011; Meyer et al. 2010). Despite the diverse negative consequences of pathological gambling, only few affected individuals seek professional help (Bischof et al. 2012). Feelings like shame and embarrassment and a limited accessibility of face-to-face treatment are important barriers to seeking support (Suurvali et al. 2009; Rockloff and Schofield 2004). Web-based interventions can help reducing these barriers, since they allow for an anonymous treatment setting and can be accessed even from remote regions (Quaglio et al. 2017; Rodda et al. 2013). Although there is robust evidence on the effectiveness of web-based psychological interventions in a variety of mental disorders (Andersson et al. 2019), a considerable lack of research exists on guided interventions targeting problem gambling (Danielsson et al. 2014; Giroux et al. 2017). So far, research was conducted on a therapist-guided program developed by the Swedish Spelinstitutet (Carlbring and Smit 2008; Carlbring et al. 2012; Castrén et al. 2013) and on another multi-week cognitive behavioral therapy (CBT) treatment (Casey et al. 2017). Their results indicate that treatment-seeking gamblers may effectively be treated online (Carlbring and Smit 2008; Carlbring et al. 2012; Casey et al. 2017).

In Germany, the Federal Centre for Health Education (BZgA) offers the prevention website www.check-dein-spiel.de (CDS, “check your gambling”). Besides information and an interactive self-test, CDS provides counseling for gamblers and their significant others. Counseling is provided via email and via the web-based program “Check Out” (CO). Email counseling (EC) targets gamblers in general and their significant others and often serves as a first contact option with professionals. As a measure of indicated prevention, CO explicitly targets individuals aiming to cease gambling. With up to 50 days of structured, individual counseling and different interactive elements, CO offers considerably more support than EC. To reduce barriers for using help, CO and EC both are free of charge and can be used fully anonymous.

To broaden the knowledge in this field of research and to provide specific empirical evidence for the counseling offers of CDS, we tested whether CO and EC effectively support individuals who want to cease gambling. Since CO and EC differ considerably in terms of accessibility, structure, user engagement and effort, a second goal was to compare the efficacy of CO and EC. We expected a greater reduction of gambling behavior and a higher increase of subjective well-being among users of both interventions compared to non-treated individuals. Moreover, we hypothesized stronger effects in CO than in EC.

## Methods

### Study Design

We conducted an open-label, purely web-based randomized controlled trial (RCT). Participants were either allocated to the web-based intervention CO, to EC or a waitlist (WL). The trial was carried out on the website https://www.check-dein-spiel.de. Individuals interested in signing up for CO were comprehensively informed about the study and were invited to participate. A PDF-file containing all relevant study details was offered for download and was submitted in the confirmation email for study participants. Persons who agreed to participate gave their informed consent by registering and checking an “I agree to participate” checkbox. Users of CO who opted not to participate in the study or who did not meet the eligibility criteria had access to the regular version of CO and were not included in any follow-up surveys.

After registration, study participants were to choose an appointment from a schedule provided by CO. By logging into the program at this appointment, they were randomized and automatically forwarded to CO, EC or were informed that they were allocated to the WL. To achieve similar group sizes, we used block randomization with 9 allocations per block. The allocation schedule was created beforehand with random generator software. Members of the waitlist group were invited to use CO after completing the first follow-up 3 months after randomization.

Follow up surveys were conducted online 3 months after randomization with all study groups and 6 and 12 months after randomization with users of CO and EC only. Each follow up participation was compensated with a shopping voucher for a major Internet-store worth 10 Euro. According to the terms and conditions of this store, it is not possible to cash out the vouchers. Five months after starting the trial, we increased the remuneration to a voucher worth 20 Euro to promote follow-up participation. The study was approved by the ethics committee of the Department of Applied Human Sciences at the University of Magdeburg-Stendal and was registered with the German Clinical Trials Register (DRKS-ID: DRKS00011569).

### Measures

Primary outcomes were the severity of problem gambling and the number of days gambled during the past 30 days. The degree of problem gambling was measured by the Problem Gambling Severity Index (PGSI; Ferris and Wynne 2001), changing the reference period from 12 to 3 months to comply with the follow-up intervals. The PGSI is a subset of 9 items of the Canadian Problem Gambling Index (CPGI) and showed good levels of reliability and validity in earlier studies (Stinchfield et al. 2007; Orford et al. 2010). Compared to the widely employed South Oaks Gambling Screen (Lesieur and Blume 1987), the PGSI is shorter and offers a continuous differentiation to describe problem gambling (Orford et al. 2010). We used the raw score of the PGSI as primary outcome. To describe the study sample, we also categorized participants with a score of at least 8 points as problem gamblers, as suggested by Ferris and Wynne (2001). To measure the gambling days, participants were asked to estimate the number of days gambled over the past 30 days. Secondary outcomes were the highest single stake during the past 30 days based on self-report and the subjective well-being of the participants as measured by the WHO-5 well-being index (Brähler et al. 2007).

With regard to process evaluation, the acceptability of CO and EC as well as the quality of the cooperation between client and counselor were measured with the Client Satisfaction Questionnaire (CSQ-8; Larsen et al. 1979; Schmidt et al. 1994) and the Working Alliance Inventory (WAI-sr; Horvath and Greenberg 1989; Wilmers et al. 2008). To gain insights on intervention usage, we furthermore tracked the number of days each intervention was used, and the time counselors spent on each case. Moreover, participants were asked at all data collection points whether they currently used other organized help offers (e.g. local counseling services, psychotherapy or support groups).

### Inclusion Criteria

To be included in the study, individuals had to be at least 18 years old and to be first time users of any counseling option of CDS. Exclusion criteria were alcohol use disorder operationalized by a score of at least 3 in the CAGE (Ewing 1984), a current diagnosis of psychotic or bipolar disorder^1^ and suicidal tendencies.^2^ Individuals who indicated suicidal tendencies were given detailed information on suitable psychosocial support offers such as telephone helplines or local institutions.

To account for possible comorbidities and circumstances of problem gamblers, inclusion criteria were rather liberal. In contrast to other studies, individuals with elevated depression symptomatology (Carlbring and Smit 2008) or legal problems (Casey et al. 2017) were not per se excluded and inclusion also was not constrained to users of certain gambling games (Bücker et al. 2018). To comply with the anonymous setting of CO and EC, the only personal information required for trial registration was a valid email-address. Prospective users were informed that it was possible to participate with an anonymous email-address.

### Interventions

#### Check Out (CO)

The web-based, structured intervention CO offers counseling by trained psychotherapists over a period of up to 50 days. CO is based on the principles of self-regulation and self-control (Kanfer 1986), the solution-focused approach (de Shazer et al. 2007) and Motivational Interviewing (Rollnick and Miller 1991). CO comprises three consecutive phases:

1. Admission takes place during a prescheduled one-to-one chat with a counselor. The chat takes 50 min and is mandatory to enter CO. It aims at clarifying the situation of the client, activating resources and establishing immediate coping strategies. In doing so, users are informed on how first steps for gambling abstinence can be realized, e.g. how to have oneself excluded in casinos, how to install filter software for online gambling and recommendations to delegate the money management to somebody else.
2. After the admission chat, the login-area of CO is activated. It contains a diary where participants are required to write down all relevant aspects of their gambling behavior over the whole duration of 50 days. The program also includes interactive exercises supporting the development of control strategies, enhancing quality of life, balancing the pros and cons of gambling, gaining an overview on debts, and developing an emergency kit for high risk situations. Once a week, participants receive detailed feedback by their counselor on their entries in the diary and the exercises. It includes general motivation to continue the diary, feedback on the current gambling activities, the psychosocial situation, possible solutions and the counseling process as such.
3. At the end of the program, clients are invited to a 30-min concluding chat, where the initial goals and the applied control strategies are reflected and goal attainment is reinforced. All participants are recommended to also contact local institutions, like addiction advice centers, support groups or, if it appeared to be indicated, psychotherapy.

#### Email Counseling (EC)

EC comprises a time-lagged message exchange between each client and his/her associated counselor. In the study, EC had the same duration and was conducted by the same counselors as CO. During the first contact, participants of EC were asked to describe their current situation as detailed as possible, to give information on their gambling activities, on the impact of gambling on their life and on their reasons for stopping gambling. This information was the basis for the first counselor’s response and the subsequent interaction.

EC did not implement any interactive or structural elements, like a diary, exercises or weekly tasks. Instead, steps on how to cope with gambling problems were outlined and discussed in the messages. For this purpose, clients were encouraged to work through information and PDF-worksheets containing “tips to overcome gambling problems” (“Tipps zur Bewältigung von Spielproblemen”) provided on CDS. Like CO, interaction in EC was based on the solution-focused approach (de Shazer et al. 2007) and Motivational Interviewing (Rollnick and Miller 1991). Since EC did not follow a predefined sequence or structure, clients were able to use it at their own pace and how often as they wished.

To ensure secure communication between client and counselor, exchange of messages took place within the password-protected login-area of CDS. Participants were notified by email when they received a message from their counselor and asked to log into the website of CDS to access the message.

### Statistical Analysis

To test the efficacy of CO and EC, we used Generalized Estimating Equations (GEE) with study group and time-factor as main effects and their interaction in each estimation model. We assumed effects on each outcome if the interaction between group and time was statistically significant. Before conducting the analyses, we tested whether variables with group differences at baseline, variables with group differences in follow-up participation and the usage of other support offers moderated the effects on each study outcome. If significant, the respective term and its interaction with study group were included in the model. Otherwise, it was not considered in the effectiveness testing. Since satisfaction and working alliance were collected only at the first follow-up, these variables were analyzed with Generalized Linear Models (GLM).

For further information, effect sizes (Cohen’s d) and the number needed to treat (NNT) including their 95%-confidence intervals were calculated. Cohen’s d was calculated by subtracting the mean change between baseline and each follow-up of one group (e.g. CO) from the mean change between baseline and each follow-up of the comparator group (e.g. WL), divided by their pooled baseline standard deviation. According to a simulation study, this approach provides better estimates of the population effect size than commonly used effect sizes calculations for repeated-measures control group designs (Morris 2008). The NNT was calculated as the reciprocal of the absolute risk reduction (ARR), and rounded up to the next integer. The ARR itself was computed using the proportion of participants per study group who abstained from gambling in the past month. This information was derived from the outcome “gambling days” at the 3-months follow-up.

All analyses were conducted following the intention-to-treat principle (ITT), including all randomized participants according to their group allocation. Missing data was estimated by multiple imputations (m = 100). To gain information on the robustness of these results, they were compared to the according results of the non-imputed dataset (completer only-analyses).

Fisher’s exact tests, one-way analyses of variance (ANOVA) or independent samples T-Tests were used to compare the study groups at baseline, to compare study participants with non-participants and to determine whether baseline measures predicted follow-up participation. These comparisons were conducted with all variables listed in Table 1 (i.e. sociodemographic variables, gambling-related variables and the usage of the intervention). Skewed distributions were log-transformed before conducting these comparisons.

**Table 1:** Participant characteristics at baseline and usage of the interventions

	CO (n = 54)	EC (n = 56)	WL (n = 57)	All participants (n = 167)
Gender, n (%)				
Female	15 (27.8%)	16 (28.6%)	16 (28.1%)	47 (28.1%)
Male	39 (72.2%)	40 (71.4%)	41 (71.9%)	120 (71.9%)
Age, mean (SD)	33.7 (10.7)	31.2 (9.1)	35.5 (11.5)	33.5 (10.6)
Educational level, n (%)				
Basic school (Hauptschule)	8 (14.8%)	10 (17.9%)	8 (14.0%)	26 (15.6%)
Middle school (Realschule)	23 (42.6%)	19 (33.9%)	23 (40.4%)	65 (38.9%)
High school (Gymnasium)	20 (37.0%)	25 (44.6%)	24 (42.1%)	69 (41.3%)
Other school	3 (5.6%)	2 (3.6%)	2 (3.5%)	7 (4.2%)
Employment status, n (%)				
Employed	35 (64.8%)	38 (67.9%)	39 (68.4%)	112 (67.1%)
In education	6 (11.1%)	8 (14.3%)	6 (10.5%)	20 (12.0%)
Unemployed	3 (5.6%)	4 (7.1%)	3 (5.3%)	10 (6.0%)
Other	10 (18.5%)	6 (10.7%)	9 (15.8%)	25 (15.0%)
Gambling behavior^a^				
Gambling prevalence, n (%)	54 (100.0%)	56 (100.0%)	56 (98.2%)	166 (99.4%)
Gambling days, mean (SD)	14.8 (8.5)	13.2 (7.0)	14.9 (9.4)	14.3 (8.4)
Problem gambling (PGSI score), mean (SD)	16.4 (4.5)	16.2 (5.1)	16.2 (4.8)	16.3 (4.8)
Problem gambling (PGSI > 7), n (%)	53 (98.1%)	54 (96.4%)	57 (100.0%)	164 (98.2%)
Highest stake (Euro), mean (SD)	583.6 (405.1)	457.6 (432.4)	510.4 (409.9)	516.4 (416.8)
Hours per day, mean (SD)	3.8 (2.2)	3.5 (2.6)	4.0 (3.0)	3.8 (2.6)
Gambling game,^a^ n (%)				
Gaming machines	43 (79.6%)	36 (64.3%)	33 (57.9%)	112 (67.1%)
Online gambling	31 (57.4%)	43 (76.8%)	34 (59.6%)	108 (64.7%)
Lotteries	10 (18.5%)	15 (26.8%)	10 (17.5%)	35 (21.0%)
Betting (offline)	5 (9.3%)	2 (3.6%)	12 (21.1%)	19 (11.4%)
Other	2 (3.7%)	9 (16.1%)	4 (7.0%)	15 (9.0%)
Utilization of other support	10 (18.5%)	12 (21.4%)	12 (21.1%)	34 (20.4%)
Well-being, mean (SD)				
WHO-5	7.4 (4.7)	8.5 (4.7)	9.2 (4.6)	8.4 (4.7)
Usage of the intervention, mean (SD)				
Days of participation	24.8 (18.9)	11.0 (13.4)	N/A	17.8 (17.7)
Time spent per user (minutes)	249.3 (103.5)	96.4 (58.4)	N/A	171.5 (113.2)

^a^During the past 30 days

The trial was powered to detect medium sized group differences (f = 0.25), which requires a total sample of n = 159 (alpha = 0.05; beta = 0.20). Data were analyzed with R 3.5.1 (R Core Team 2018).

## Results

### Flow of Participants

**Fig. 1:**
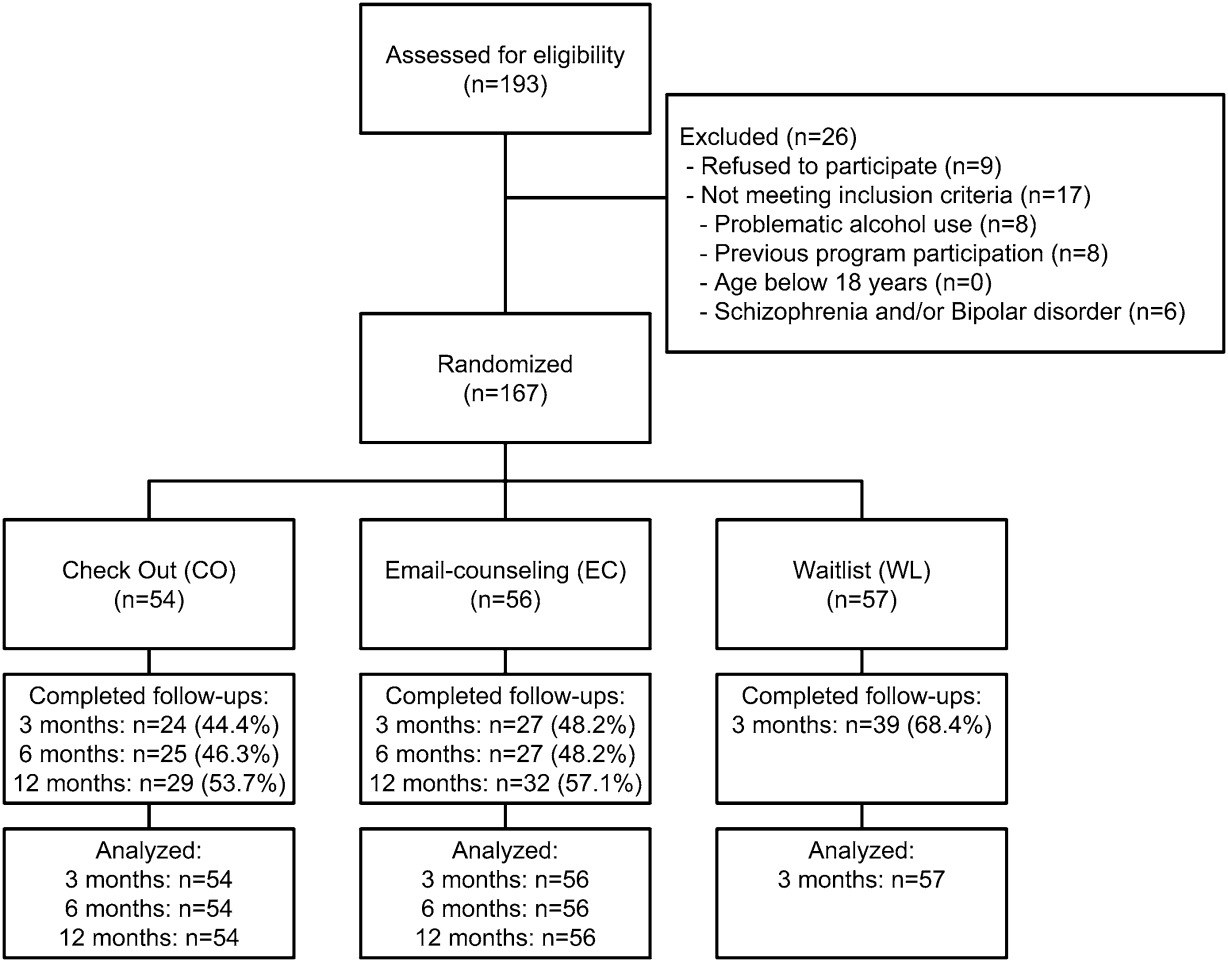
CONSORT flow diagram of participants

During the study, 193 individuals accessed the baseline questionnaire of CDS and were assessed for eligibility (Fig. 1). 26 individuals did not take part at the study, because they did not meet inclusion criteria (n = 17) or refused to participate (n = 9). The randomization of the 167 participants resulted in similar-sized study groups. 90 individuals provided data at the first follow-up, 52 at the second, and 61 participants filled out the last follow-up survey 12 months after randomization, resulting in follow-up rates of 53.9%, 47.3% and 55.5% respectively. Participants who took part at the follow-ups used their intervention longer than those who were lost to follow-up (*t*(108) = 3.266, *p *= 0.001; Follow-up participants: M = 23.0 days, SD = 17.0 days; Follow-up non-participants: M = 14.3 days, SD = 17.5 days; *d *= 0.50). Beyond that more time was spent on their cases than on those who did not take part at any follow-up (*t*(108) = 2.382, *p *= 0.019; Follow-up participants: M = 196.2 min, SD = 112.8 min; Follow-up non-participants: M = 155.0 min, SD = 111.3 min; *d *= 0.37). In contrast, the comparison with individuals who used CO without taking part at the study (n = 26, see Fig. 1) did not reveal any significant differences.

After raising the remuneration for the follow-up participation, the attendance rate at the 3- and 6-months follow-ups increased from 48.4 to 68.9% (3 months) and from 41.0 to 50.7% (6 months). Adherence to the 12-months follow-up was not affected, since the higher remuneration was introduced before the first 12-months-follow-up was conducted.

### Sample Characteristics

Baseline characteristics and intervention usage of the study participants are shown in Table 1. The majority of participants were male (71.9%) and had an above average educational level with 41.3% attending or having successfully finished the highest German secondary school type (“Gymnasium”; Federal Statistical Office of Germany 2019). As expected, the gambling behavior of almost all participants (98.2%) was problematic according to their PGSI score. Almost all participants gambled in the 30 days prior to registration (99.4%). In that period, participants gambled on 14.3 days, with gaming machines (67.1%) and online gambling (64.7%) named most frequently. Approximately one in five participants (20.4%) was utilizing some sort of organized support for their problem gambling, mainly local addiction counseling (11.4%), outpatient psychotherapy (9.6%) or a support group (3.0%).

Except for between-group differences in the highest stake (*F*(2, 164) = 3.192; *p *= 0.044), the usage of gaming machines (*p *= 0.042) and offline-betting (*p *= 0.014), randomization resulted in similar groups.

With an average of 24.8 versus 11.0 days, CO was used more than twice as long as EC (*t*(108) = 2.983; *p *= 0.004; *d *= 0.84). Moreover, counselors spent considerably more time per CO-client than per EC-client (*t*(108) = 8.181; *p *< 0.001; *d *= 1.82).

According to the analysis plan, the following variables were tested as possible moderators in the effective analyses: The highest stake, the usage of gaming machines, the usage of offline-betting, the days of intervention participation, utilization of other support and the counselor’s time spent per user. However, since none of these moderated the effects of group assignment on any outcome (*p *≥ 0.052), none was included in the effectiveness analyses.

### Treatment Effects

Descriptive statistics and effect sizes for the outcome variables are shown in Table 2. In Table 3, the results of the corresponding statistical tests are reported. Compared to WL at the 3 months follow-up, significant and moderate to strong effects in favor of CO were found in all outcome measures. The highest mean differences between CO and WL were observed in the severity of problem gambling (*d *= 0.91), followed by the general well-being (*d *= 0.70), the number of gambling days (*d *= 0.59) and the highest stake during the past 30 days (*d *= 0.55; see Tables 2, 3). These results were similar to those of the non-imputed dataset (see “Appendix”). The mean effect size of all four outcomes amounts to *d *= 0.69.

**Table 2:** Descriptive statistics and effect sizes for the outcomes

	CO (n = 54)^a^	EC (n = 56)^a^	WL (n = 57)^a^	CO versus EC *d* [95%-CI]^b^	CO versus WL *d* [95%-CI]^b^	EC versus WL *d* [95%-CI]^b^
Gambling days^c^						
Baseline	14.8 (8.5)	13.2 (7.0)	14.9 (9.4)			
3 months	2.4 (4.2)	3.5 (5.4)	7.9 (8.8)	0.35 [− 0.03 to 0.73]	0.59 [0.20 to 0.97]	0.31 [− 0.07 to 0.68]
6 months	4.0 (6.8)	4.8 (6.5)		0.30 [− 0.08 to 0.68]		
12 months	3.3 (5.0)	3.0 (4.5)		0.17 [− 0.21 to 0.55]		
Problem gambling (PGSI)						
Baseline	16.4 (4.5)	16.2 (5.1)	16.2 (4.8)			
3 months	7.7 (6.4)	8.1 (7.0)	11.8 (7.0)	0.12 [− 0.25 to 0.50]	0.91 [0.52 to 1.31]	0.74 [0.36 to 1.13]
6 months	5.7 (6.8)	8.2 (7.6)		0.56 [0.17 to 0.94]		
12 months	5.1 (6.2)	7.4 (7.3)		0.52 [0.14 to 0.90]		
Highest stake (Euro)^c^						
Baseline	583.6 (405.1)	457.6 (432.4)	510.4 (409.9)			
3 months	97.0 (187.9)	128.7 (179.3)	249.4 (262.3)	0.37 [− 0.01 to 0.75]	0.55 [0.17 to 0.93]	0.16 [− 0.21 to 0.53]
6 months	139.7 (327.9)	130.3 (214.1)		0.28 [− 0.10 to 0.66]		
12 months	125.0 (248.4)	229.9 (433.8)		0.55 [0.16 to 0.93]		
Well-being (WHO-5)						
Baseline	7.4 (4.7)	8.5 (4.7)	9.2 (4.6)			
3 months	13.9 (5.1)	13.7 (5.0)	12.4 (5.2)	0.28 [− 0.10 to 0.66]	0.70 [0.32 to 1.09]	0.43 [0.05 to 0.80]
6 months	13.7 (5.5)	13.9 (5.9)		0.18 [− 0.20 to 0.56]		
12 months	15.6 (4.9)	13.4 (5.3)		0.68 [0.29 to 1.07]		
Working alliance (WAI-sr)						
3 months	46.9 (8.8)	40.4 (9.7)		0.70 [0.31 to 1.09]		
Treatment satis-faction (CSQ-8)						
3 months	25.7 (4.6)	23.5 (4.5)		0.49 [0.10 to 0.87]		

^a^Mean (SD)

^b^Positive effect sizes refer to outcomes beneficial to the first-mentioned study group

^c^During the past 30 days

**Table 3:** Parameter estimates for the outcomes

	CO versus EC		CO versus WL		EC versus WL	
	Beta [95%-CI]	*p* value	Beta [95%-CI]	*p* value	Beta [95%-CI]	*p* value
Gambling days^a^						
3 months	0.51 [− 0.35 to 1.37]	0.247	1.19 [0.47 to 1.92]	0.001	0.68 [0.05 to 1.32]	0.035
6 months	0.29 [− 0.48 to 1.05]	0.460				
12 months	0.02 [− 0.76 to 0.81]	0.958				
Problem gambling (PGSI)						
3 months	0.07 [− 0.36 to 0.50]	0.756	0.45 [0.07 to 0.83]	0.023	0.38 [0.06 to 0.71]	0.022
6 months	0.38 [− 0.13 to 0.90]	0.146				
12 months	0.40 [− 0.13 to 0.92]	0.138				
Highest stake (Euro)^a^						
3 months	0.55 [− 0.42 to 1.52]	0.267	1.12 [0.26 to 1.99]	0.012	0.57 [− 0.09 to 1.24]	0.092
6 months	0.18 [− 0.89 to 1.25]	0.742				
12 months	0.90 [− 0.34 to 2.15]	0.156				
Well-being (WHO-5)						
3 months	− 0.15 [− 0.41 to 0.11]	0.255	− 0.33 [− 0.58 to − 0.08]	0.011	− 0.18 [− 0.42 to 0.06]	0.141
6 months	− 0.12 [− 0.36 to 0.13]	0.359				
12 months	− 0.28 [− 0.52 to − 0.04]	0.020				
Working alliance (WAI-sr)						
3 months	− 0.15 [− 0.28 to − 0.03]	0.019				
Treatment satis-faction (CSQ-8)						
3 months	− 0.09 [− 0.20 to 0.02]	0.109				

Between-group comparisons were conducted by analyzing the interaction of study group and time. To test the effects on WAI-sr and CSQ-8, the main effect of study group (CO and EC only) was analyzed

^a^During the past 30 days

In the comparison of EC and WL, we observed significant beta coefficients and effect sizes only in the severity of problem gambling (*d *= 0.74; see Tables 2, 3). No significant group differences between CO and EC were observed at first follow-up. With the exception of the wellbeing-score at the 12-months-follow-up in favor of CO (*d *= 0.68), no other effect differences were detected either. Significant differences were however found in the working alliance, with clients of CO reporting a stronger working alliance than users of EC (*d *= 0.70). In treatment satisfaction, no significant difference was observed. These results were confirmed by the results of the non-imputed dataset (see “Appendix”).

Significant and strong effects between baseline and the 12 months-follow up indicate substantial and lasting reductions of gambling behavior both in CO and in EC (see Table 4). In CO, the strongest within-group effects were found in the PGSI-score with a reduction from 16.4 points at baseline to 5.1 points 1 year later (*d *= 2.48). In EC, the largest reductions were found in the gambling frequency (*d *= 1.72).

**Table 4:** Within-group effects in CO and EC

	CO			EC		
	*d* [95%-CI]	*t* (106)	*p* value	*d* [95%-CI]	*t* (110)	*p* value
Gambling days^a^	1.34 [0.92 to 1.76]	6.96	< 0.001	1.44 [1.02 to 1.86]	7.64	< 0.001
Problem gambling (PGSI)	2.48 [1.97 to 2.98]	12.86	< 0.001	1.72 [1.28 to 2.15]	9.08	< 0.001
Highest stake (Euro)^a^	1.12 [0.71 to 1.53]	5.84	< 0.001	0.52 [0.14 to 0.90]	2.77	0.003
Well-being (WHO-5)	1.73 [1.28 to 2.17]	8.96	< 0.001	1.04 [0.64 to 1.44]	5.51	< 0.001

Positive effect sizes refer to beneficial outcomes

^a^During the past 30 days

At the 3 months follow-up, 24 of 54 (44.4%) of CO-participants had abstained from gambling, as compared to 13 of 57 (22.8%) in the WL, resulting in a number needed to treat of NNT = 5 (CI 2.58–22.31). 22 of all 56 users of EC (39.3%) did not gamble in the 30 days prior the 3 months follow-up, resulting in an NNT of 7 (CI 3.00–∞).

## Discussion

In this study we examined whether the web-based intervention CO and EC of CDS effectively reduce problem gambling and increase subjective well-being of treatment-seeking gamblers. Compared to individuals on a waitlist, users of CO reduced their gambling with medium to strong effects and improved their subjective well-being in a similar size. Evidence on the efficacy of EC is less strong, since individuals who used EC achieved robust effects in only one outcome, problem gambling.

Although effects in CO tend to be stronger than in EC, none of these differences were significant. This may be attributed to the limited sample size of the trial, which was powered only to detect at least medium sized effects. Compared to EC, clients of CO however reported a significantly stronger working alliance with their counselor, and used the intervention more than twice as long. This suggests that users of CO feel more affiliated to their intervention than individuals who use EC. One reason for that may be the immediate interaction in the admission chat which explicitly aims at establishing a viable working relationship. Moreover, the interactive elements of CO and its repetitive routines (diary, weekly feedbacks) presumably had an engaging effect on the users, and motivated them to work on their goals. The absence of any comparable modules in EC might in contrast be perceived as a lack of guidance. To increase adherence and efficacy, EC should therefore probably have been complemented with additional elements like e.g. weekly tasks or exercises, known from other email-based treatments (Ruwaard et al. 2007; Vernmark et al. 2010).

The results of CO are consistent with outcomes of two other RCTs on web-based interventions for treatment-seeking problem gamblers (Carlbring and Smit 2008; Casey et al. 2017). In these studies, CBT-based treatments with six (Casey et al. 2017) respectively eight (Carlbring and Smit 2008) weekly modules were tested. The latter intervention also included weekly telephone calls lasting approx. 15 min each. Compared to individuals on a waitlist at post-intervention, participants of these interventions benefitted in various outcomes, and maintained these improvements over time. CO achieves a lower composite effect (*d *= 0.69) than the intervention tested by Carlbring and Smit (*d *= 0.83). This however might be attributed to the additional voice contacts provided in that intervention. Casey et al. do not report effect sizes.

The number of persons needed to be treated (NNT) in CO to achieve gambling abstinence is five. This value falls within the range of web-based interventions targeting substance abuse (no previous study on web-based Interventions for problem gamblers reports NNTs so far). In a therapist-guided intervention for alcohol users, the NNT for treatment response was also five (Blankers et al. 2011). In a Meta-Analysis, NNTs between 9 and 26 for achieving abstinence or reduction were reported for web-based interventions targeting substance use (Rogers et al. 2017). Another Meta-Analysis reports an average NNT of 10 for therapist-guided interventions for cannabis users (Tait et al. 2013).

With a reduction from 100% by registration to 55.6% after 3 months, gambling prevalence in CO is reduced considerably. Traditional outpatient counseling centers in Germany show reductions in gambling prevalence from 72.2% in the month prior treatment to 43.6% in the last month of treatment (Institut für Therapieforschung 2019). Results of our trial therefore suggest, that CO can effectively complement traditional outpatient counseling centers in Germany.

Since the time spent per client and the duration of participation were not associated with any treatment outcome, it may seem considerable to shorten the maximum duration of CO. Other research also suggests, that adequate treatment effects may be achieved within a shorter intervention and with less time spent by the counselors (Casey et al. 2017; Jonas et al. 2018). This assumption should however be backed by further research. Potential moderators or mediators of sustainable treatment effects should also be included in those studies.

Since the educational level of participants was relatively high, further research should also investigate ways to reach lower educated individuals and other vulnerable subgroups of gamblers. To provide further empirical evidence for CO, a comparison of CO with treatment as usual (e.g. local counseling services) would also be valuable.

### Limitations

This study has several limitations. Common to web-based trials (e.g. Casey et al. 2017; Bücker et al. 2018), follow-up attrition was considerable. Although we imputed missing data and included potential confounders in the analyses, validity of results might therefore be reduced. To reduce missing data, we decided to increase the remuneration for follow-up participation which indeed raised participation rates significantly. To address ethical concerns, we opted to incentivize participants with shopping vouchers instead of cash.

Moreover, as all purely web-based trials, we relied on self-reported data. Since gambling has strong effects on cognitive appraisal and perception, gambling-related data may therefore be biased. Participants might also have understated their gambling activities to reduce feelings of shame and guilt. Since the anonymous setting of this trial presumably promoted disinhibition and an open expression of emotions (Suler 2004), this problem however is probably mitigated to some extent.

Another possible limitation of the study is the implementation of a waitlist as control condition, since this approach may overestimate treatment effects. In a network meta-analysis including RCTs on CBT interventions for major depression, Furukawa and colleagues (2014) found that study participants on a waitlist performed worse than individuals who did not receive treatment even after a waiting period (i.e. an actual no treatment condition). Due to the ethical constraints associated with de facto no treatment conditions, we however did not consider this type of control when designing our study. At this, our study corresponds with a wide array of RCTs in this and neighboring research fields (see Danielsson et al. 2014; Giroux et al. 2017 for reviews).

Since the counseling team was aware of the study hypotheses, we furthermore cannot rule out that this somehow affected the study results. Although there was no such indication when adherence to counseling standards was monitored, expectations or preferences of the counseling staff may still have affected the results in either way.

### Conclusions

According to the results, CO helps treatment-seeking individuals to sustainably reduce their gambling and to increase their general well-being. These results correspond to the other few studies in this field of research. CO suits treatment-seeking gamblers presumably better than EC, since the effects of CO tend to be stronger and include more outcomes. Reasons for the superiority of CO possibly are the more engaging program structure and elements, as well as the closer interaction between client and counselor. EC therefore should mainly be used as an easy-to-reach counseling option for first-time support seekers. Individuals aiming to quit gambling should be referred to CO.

## Appendix

The following tables display the results of the non-imputed dataset (completer only-analyses).

See Tables 5, 6 and 7.

**Table 5:** Descriptive statistics and effect sizes for the outcomes

	CO (n = 54)^a^	EC (n = 56)^a^	WL (n = 57)^a^	CO versus EC *d* [95%-CI]^b^	CO versus WL *d* [95%-CI]^b^	EC versus WL *d* [95%-CI]^b^
Gambling days^c^						
Baseline	14.8 (8.5)	13.2 (7.0)	14.9 (9.4)			
3 months	2.1 (4.2)	3.7 (6.1)	8.0 (8.9)	0.42 [− 0.15 to 0.99]	0.64 [0.11 to 1.18]	0.31 [− 0.20 to 0.81]
6 months	3.9 (7.7)	4.8 (6.4)		0.32 [− 0.24 to 0.87]		
12 months	3.3 (5.3)	2.9 (4.2)		0.16 [− 0.35 to 0.67]		
Problem gambling (PGSI)						
Baseline	16.4 (4.5)	16.2 (5.1)	16.2 (4.8)			
3 months	7.1 (6.4)	8.0 (7.0)	11.5 (7.0)	0.23 [− 0.34 to 0.79]	0.95 [0.41 to 1.50]	0.68 [0.17 to 1.19]
6 months	5.2 (6.9)	8.7 (7.5)		0.76 [0.19 to 1.34]		
12 months	5.1 (6.4)	7.3 (7.0)		0.48 [− 0.04 to 1.00]		
Highest stake (Euro)^c^						
Baseline	583.6 (405.1)	457.6 (432.4)	510.4 (409.9)			
3 months	93.3 (213.6)	126.2 (171.8)	251.0 (271.2)	0.38 [− 0.19 to 0.95]	0.56 [0.03 to 1.09]	0.17 [− 0.33 to 0.67]
6 months	125.7 (397.6)	131.4 (200.6)		0.31 [− 0.24 to 0.87]		
12 months	95.1 (197.5)	233.4 (452.2)		0.63 [0.10 to 1.15]		
Well-being (WHO-5)						
Baseline	7.4 (4.7)	8.5 (4.7)	9.2 (4.6)			
3 months	14.2 (5.2)	14.1 (4.9)	12.6 (5.3)	0.24 [− 0.31 to 0.80]	0.72 [0.20 to 1.24]	0.47 [− 0.03 to 0.98]
6 months	13.5 (5.3)	14.2 (6.0)		0.07 [− 0.48 to 0.62]		
12 months	15.6 (4.7)	13.4 (5.0)		0.67 [0.15 to 1.20]		
Working alliance (WAI-sr)						
3 months	46.4 (9.1)	39.5 (11.1)		0.66 [0.10 to 1.23]		
Treatment satis-faction (CSQ-8)						
3 months	25.6 (4.9)	23.3 (5.0)		0.46 [− 0.10 to 1.02]		

^a^Mean (SD)

^b^Positive effect sizes refer to outcomes beneficial to the first-mentioned study group

^c^During the past 30 days

**Table 6:** Parameter estimates for the outcomes

	CO versus EC		CO versus WL		EC versus WL	
	Beta [95%-CI]	*p* value	Beta [95%-CI]	*p* value	Beta [95%-CI]	*p* value
Gambling days^a^						
3 months	0.73 [− 0.19 to 1.65]	0.118	1.32 [0.55 to 2.10]	0.001	0.59 [− 0.07 to 1.25]	0.078
6 months	0.30 [− 0.57 to 1.18]	0.496				
12 months	0.07 [− 0.65 to 0.78]	0.859				
Problem gambling (PGSI)						
3 months	0.13 [− 0.29 to 0.55]	0.542	0.49 [0.13 to 0.86]	0.008	0.37 [0.04 to 0.69]	0.030
6 months	0.48 [− 0.08 to 1.04]	0.090				
12 months	0.40 [− 0.14 to 0.95]	0.146				
Highest stake (Euro)^a^						
3 months	0.55 [− 0.41 to 1.50]	0.262	1.11 [0.21 to 2.02]	0.016	0.57 [− 0.06 to 1.19]	0.076
6 months	0.22 [− 1.09 to 1.52]	0.746				
12 months	1.10 [0.11 to 2.09]	0.029				
Well-being (WHO-5)						
3 months	− 0.18 [− 0.42 to 0.07]	0.163	− 0.34 [− 0.58 to − 0.11]	0.005	− 0.17 [− 0.41 to 0.07]	0.164
6 months	− 0.11 [− 0.37 to 0.14]	0.392				
12 months	− 0.30 [− 0.54 to − 0.07]	0.011				
Working alliance (WAI-sr)						
3 months	− 0.16 [− 0.24 to − 0.08]	< 0.001				
Treatment satis-faction (CSQ-8)						
3 months	− 0.10 [− 0.20 to 0.01]	0.088				

Between-group comparisons were conducted by analyzing the interaction of study group and time. To test the effects on WAI-sr and CSQ-8, the main effect of study group (CO and EC only) was tested

^a^During the past 30 days

**Table 7:** Within-group effects in CO and EC

	CO			EC		
	*d* [95%-CI]	*t* (106)	*p* value	*d* [95%-CI]	*t* (110)	*p* value
Gambling days^a^	1.34 [0.83 to 1.84]	5.80	< 0.001	1.45 [0.96 to 1.94]	6.54	< 0.001
Problem gambling (PGSI)	2.47 [1.87 to 3.07]	10.73	< 0.001	1.75 [1.23 to 2.26]	7.88	< 0.001
Highest stake (Euro)^a^	1.20 [0.70 to 1.69]	5.20	< 0.001	0.51 [0.07 to 0.96]	2.32	0.011
Well-being (WHO-5)	1.72 [1.19 to 2.25]	7.48	< 0.001	1.05 [0.58 to 1.52]	4.73	< 0.001

Positive effect sizes refer to beneficial outcomes

^a^During the past 30 days

## Footnotes

1 Current psychotic or bipolar disorder was measured by the following self-developed item: “Were you recently diagnosed with any of the following disorders? (1) Schizophrenia/psychotic disorder/(2) Bipolar disorder/(3) I was not diagnosed with any of these disorders” Answering “yes” to either (1) or (2) or both lead to exclusion from the study.

2 Suicidal tendencies were measured by the following self-developed items: “(1) Did you undertake any suicide attempt in recent years?/(2) Do you currently wish to attempt suicide?” Answering “yes” to either or both (1) or (2) lead to exclusion from the study.
