# Correction to: Web-Based Intervention and Email‑Counseling for Problem Gamblers: Results of a Randomized Controlled Trial

**DOI:** 10.1007/s10899-019-09914-4

**Published:** 2019-11-27

**Authors:** Benjamin Jonas, Fabian Leuschner, Anna Eiling, Christine Schoelen, Renate Soellner, Peter Tossmann

**Affiliations:** 1 Delphi - Gesellschaft für Forschung, Beratung und Projektentwicklung mbH, Kaiserdamm 8, 14057 Berlin, Germany; 2 Federal Centre for Health Education (BZgA), Cologne, Germany; 3 University of Hildesheim, Hildesheim, Germany

## Correction to: Journal of Gambling Studies 10.1007/s10899-019-09883-8

The article “Web-Based Intervention and Email-Counseling for Problem Gamblers: Results of a Randomized Controlled Trial”, written by Benjamin Jonas, Fabian Leuschner, Anna Eiling, Christine Schoelen, Renate Soellner and Peter Tossmann, was originally published electronically on the publisher’s internet portal on 27 September 2019 without open access. With the author(s)’ decision to opt for Open Choice, the copyright of the article changed on 27 November 2019 to © The Author(s) 2019 and the article is forthwith distributed under a Creative Commons Attribution 4.0 International License (https://creativecommons.org/licenses/by/4.0/), which permits use, sharing, adaptation, distribution and reproduction in any medium or format, as long as you give appropriate credit to the original author(s) and the source, provide a link to the Creative Commons licence, and indicate if changes were made. The original article has been corrected.

